# Social inequalities in multimorbidity, frailty, disability, and transitions to mortality: a 24-year follow-up of the Whitehall II cohort study

**DOI:** 10.1016/S2468-2667(19)30226-9

**Published:** 2019-12-16

**Authors:** Aline Dugravot, Aurore Fayosse, Julien Dumurgier, Kim Bouillon, Tesnim Ben Rayana, Alexis Schnitzler, Mika Kivimaki, Séverine Sabia, Archana Singh-Manoux

**Affiliations:** a Inserm U1153, Epidemiology of Ageing and Neurodegenerative Diseases, Université de Paris, Paris, France; b Cognitive Neurology Center, Hôpitaux Universitaires Saint-Louis, Lariboisière, Fernand-Widal, Assistance Hôpitaux Publique de Paris, Université de Paris, Paris, France; c Département d'Information Médicale, Centre Hospitalier de Saint-Brieuc, Saint-Brieuc, France; d Department of Epidemiology and Public Health, University College London, London, UK

## Abstract

**Background:**

Social inequalities in mortality persist in high-income countries with universal health care, and the mechanisms by which these inequalities are generated remain unclear. We aimed to examine whether social inequalities were present before or after the onset of adverse health conditions (multimorbidity, frailty, and disability).

**Methods:**

Our analysis was based on data from the ongoing Whitehall II cohort study, which enrolled British civil servants aged 35–55 years in 1985–88. Participants were assessed for three indicators of socioeconomic status (education, occupational position, and literacy) at age 50 years. Participants underwent clinical examinations (in 2002–04, 2007–09, 2012–13, and 2015–16) for assessment of frailty (two or more of low physical activity, slow walking speed, poor grip strength, weight loss, and exhaustion) and disability (two or more difficulties in bathing, dressing, going to the toilet, transferring, feeding, and walking). In addition, electronic health records were used to assess the incidence of multimorbidity (two or more of diabetes, coronary heart disease, stroke, chronic obstructive pulmonary disease, depression, arthritis, cancer, dementia, and Parkinson's disease) and mortality. In analyses adjusted for sociodemographic factors, we used multistate models to examine social inequalities in transitions from healthy state to adverse health conditions and subsequently to mortality.

**Findings:**

Of 10 308 individuals in the Whitehall II study cohort, 6425 had relevant data available at 50 years and to the end of follow-up on Aug 31, 2017, and were included in our analysis. Participants were followed up for a median of 23·6 years (IQR 19·6–28·9). 1694 (26·4%) of 6425 participants developed multimorbidity, 1733 (27·0%) became frail, 692 (10·8%) had a disability, and 611 (9·5%) died. Multimorbidity (hazard ratio [HR] 4·12 [95% CI 3·41–4·98]), frailty (HR 2·38 [95% CI 1·93–2·93]), and disability (HR 1·73 [95% CI 1·34–2·22]) were associated with increased risk of mortality; these associations were not modified by socioeconomic status. In multistate models, occupation was the socioeconomic status indicator that was most strongly associated with inequalities in the transition from healthy state to multimorbidity (HR 1·54 [95% CI 1·37–1·73]), to frailty (HR 2·08 [95% CI 1·85–2·33]), and to disability (HR 1·44 [95% CI 1·18–1·74]). Socioeconomic status indicators did not affect transitions to mortality in those with multimorbidity, frailty, or disability.

**Interpretation:**

Socioeconomic status affects the risk of multimorbidity, frailty, and disability, but does not affect the risk of mortality after the onset of these adverse health conditions. Therefore, primary prevention is key to reducing social inequalities in mortality. Of the three adverse health conditions, multimorbidity had the strongest association with mortality, making it a central target for improving population health.

**Funding:**

UK Medical Research Council; National Institute on Aging, National Institutes of Health; British Heart Foundation.

## Introduction

The ageing of populations worldwide highlights the importance of understanding drivers of health at older ages. Data on morbidity trends at older ages show continuing increases in the prevalence of chronic diseases, with some evidence of a decrease in the prevalence of functional limitations.^1^ The risk of mortality is higher in those with chronic disease^2^ and those with functional limitations as assessed by frailty^3^ or disability.^4^ In addition, social inequalities in mortality persist,^5^ with data on trends from some high-income countries, including England^6^ and the USA,^7^ indicating a widening of these inequalities. The extent to which adverse health conditions in the course of ageing are generators of inequalities in mortality remains unknown. Research in this domain is piecemeal, in that studies generally examine inequalities in morbidity and mortality, without considering the social patterning in progression from adverse health conditions to mortality.

The aim of this study was to examine whether social inequalities in mortality are generated before or after the onset of adverse health conditions, such as multimorbidity, frailty, and disability, in participants aged 50 years at the start of a median follow-up of 23·6 years. We used multistate models that allowed estimation of the role of socioeconomic status in the transition from a healthy state to adverse health conditions and the subsequent transition to mortality. A further aim was to identify key indicators of socioeconomic status that shape the risk of multimorbidity, frailty, disability, and mortality. Education and occupation are widely used indicators of socioeconomic status but other measures might be more salient for health at older ages. Longer lifespans and the increasing complexity of managing multiple health conditions require that we also consider measures such as health literacy, defined as the capacity to obtain, process, and understand the health information needed to make appropriate health decisions.^8^ We used these three socioeconomic status indicators (education, occupation, and literacy) to examine inequalities in transitions to mortality via multimorbidity, frailty, and disability to identify prevention targets that could reduce social inequalities in mortality.

**Research in context**

**Evidence before this study**

We searched PubMed for publications until Sept 25, 2019, using the Medical Subject Headings search terms “inequalities”, “socioeconomic status (SES)”, “ageing”, “frailty”, “multimorbidity”, “disability”, “morbidity”, and “mortality”. Strong evidence shows that social inequalities exist in multimorbidity, frailty, disability, and mortality, with some data over the past 10 years showing increases in inequalities in life expectancy in some high-income countries. Although studies have investigated the role of mediators in explaining social inequalities, we did not find studies that examined whether socioeconomic factors play a role in the incidence of adverse health conditions (multimorbidity, frailty, or disability) and their progression to mortality. Separate studies have shown multimorbidity, frailty, or disability to increase risk of mortality, but the three adverse health conditions have not been examined in the same study population.

**Added value of this study**

Our analysis of the temporal progression from a healthy state at age 50 years to adverse health conditions (multimorbidity, frailty, disability) and subsequent mortality over a median follow-up of 23·6 years suggests that socioeconomic status affects the risk of multimorbidity, frailty, and disability, but it does not affect the risk of mortality after the onset of these conditions. Another key finding is the strong association between multimorbidity and mortality, with the strength of this association being similar across socioeconomic status groups. In individuals without multimorbidity, frailty, and disability, we found evidence of social inequalities in mortality, primarily due to deaths from cancer. The strength of our analysis is the use of multistate models to show transitions from a healthy state to adverse health conditions and subsequent mortality in a single analytic framework, which allows us to examine how socioeconomic factors shape health trajectories.

**Implications of all the available evidence**

Social inequalities exist in the transition from healthy state to adverse health conditions, but not in the transition from adverse health condition to mortality. Therefore, primary prevention, before the onset of multimorbidity, frailty, or disability, will be important in reducing social inequalities in mortality.

## Methods

### Study design and participants

The Whitehall II study is an ongoing cohort study that was established in 1985 to investigate the role of socioeconomic circumstances for health by following up a cohort of 10 308 British civil servants (6895 men and 3413 women) who were aged 35–55 years in 1985–88.^9^ All participants responded to a comprehensive questionnaire and underwent a uniform, structured, clinical evaluation at baseline and approximately every 5 years. Participant consent and research ethics approval were renewed at each contact; the most recent approval was by the NHS London Harrow Research Ethics Committee (reference number 85/0938).

#### Indicators of socioeconomic status

Occupational position at age 50 years was available from records of British Civil Service employment grade, a comprehensive measure that reflects education, occupational status, and income. Positions are categorised as high (administrative grades), intermediate (professional or executive grades), or low (clerical or support grades).

Highest attained education was categorised as high (university or higher degree), intermediate (higher secondary school), or low (lower secondary school or less).

Literacy at age 50 years was assessed using a vocabulary test, as recommended by Kobayashi and colleagues.^10^ We used the Mill Hill vocabulary test, consisting of a list of 33 stimulus words ordered by increasing difficulty, with the person required to choose the meaning of each word from six response choices.

#### Adverse health conditions

Participants were followed up to assess the occurrence of multimorbidity, frailty, or disability, which are partially overlapping adverse health conditions. Individuals were defined as having each of these adverse health conditions if two or more of the condition-specific criteria were met. This is the standard definition of multimorbidity but not frailty and disability; therefore, sensitivity analyses were done to ensure that results were not affected by this choice.

Multimorbidity was defined as having two or more of nine specific chronic diseases, which are the leading causes of death in high-income countries. Data on these chronic diseases were ascertained from multiple sources: clinical examinations in the study and linkage to electronic health records using National Health Service (NHS) identification numbers; and four national databases, including the national hospital episode statistics database that contains inpatient and outpatient data, the Mental Health Services Data Set that contains inpatient and outpatient data in addition to data on care in the community, the cancer registry, and the mortality register.

The nine chronic conditions considered were the following (patients needed to meet at least one of the criteria in parentheses): diabetes (fasting glucose ≥7·0 mmol/L, reported doctor-diagnosed diabetes, use of diabetes medication, International Classification of Diseases version 10 [ICD-10] codes E10–14), coronary heart disease (12-lead resting electrocardiogram recording, ICD-10 codes I20–25, procedures K40–49, K50, K75, U19), stroke (MONICA-Ausburg stroke questionnaire, ICD-10 codes I60–64), chronic obstructive pulmonary disease (ICD-10 codes J41–44), depression (use of antidepressants, ICD-10 codes F32–33), arthritis (self-report of longstanding illness, ICD-10 codes M15–19), cancer (cancer registry with malignant cancer, ICD-10 codes C00–C97 to include colorectal, lung, breast, prostate, and smoking-related cancers and melanoma skin cancers), dementia (ICD-10 codes F00–03, F05·1, G30, G31), and Parkinson's disease (ICD-10 code G20).

Frailty was measured at clinical examinations (2002–04, 2007–09, 2012–13, and 2015–16) using the Fried frailty scale. The threshold for each criterion was based on the original frailty score.^11^ This strategy allows comparison of findings across studies, as opposed to the use of thresholds based on the distribution in the study population being examined. The criteria for frailty included low physical activity, slow walking speed, poor grip strength, weight loss, and exhaustion; details of these criteria are provided in the appendix (p 2). Participants were classified as frail if they met at least two of the frailty criteria.

To assess disability, in 2002–04, 2007–09, 2012–13, and 2015–16 a modified version of the Katz Index of Independence in Activities of Daily Living was included in the study questionnaire to measure disability.^12^ Disability was defined as reporting difficulty (yes or no) in performing two or more of six Activities of Daily Living: bathing, dressing, going to the toilet, transferring, feeding, and walking.

#### Mortality

Our primary outcome was death from any cause. Mortality data until Aug 31, 2017, were drawn from the UK national mortality register (National Health Services Central Registry). The tracing exercise was done using the NHS identification number of each participant.

### Statistical analysis

Data on socioeconomic status and covariates (age, sex, ethnicity, and marital status at 50 years) were extracted from the wave of data collection when the participant was aged 50 years (plus or minus 5 years) and free of adverse health conditions. Participants were followed up from age 50 years until the record of death or Aug 31, 2017, whichever came first. There was no evidence of sex differences (all p>0·05) in the association between socioeconomic status indicators and adverse health conditions (multimorbidity, frailty, and disability), leading us to combine men and women in the analysis. Because education and occupation were measured on three-point scales, literacy was also categorised into three equal groups. The correlation between these three indicators was assessed using the κ statistic. After verification for linearity, categories of socioeconomic status indicators were recoded (0, 0·5, and 1·0) so that when entered as a continuous variable, the reported hazard ratio [HR] corresponded to the increased risk in those in the lowest socioeconomic group compared with the highest socioeconomic group. All analyses were done on transitions, first using survival analysis and then with multistate models.

For the survival analysis (two possible states), proportional hazards models were used to examine the association of socioeconomic status (education, occupation, literacy) with adverse health conditions and mortality in separate models. We then examined the association of time-varying multimorbidity, frailty, disability (separate models) with subsequent mortality. These analyses were done in the total sample, and an interaction term was used to assess whether this association differed according to socioeconomic status. We repeated these analyses using time-varying, total number of adverse health conditions as the exposure (0, 1, 2, or 3).

Further analyses were done with weighted multistate models (three possible states) with a semi-Markov model (Weibull distribution)^13^ to estimate the role of socioeconomic status in transitions from (A) a healthy state to an adverse health condition (multimorbidity, frailty, or disability); (B) a healthy state to death in those without an adverse health condition; and (C) from the adverse health condition to death. Because transition C was underpowered when the adverse health outcomes were considered separately, we did sensitivity analyses in which any of the three adverse health conditions were included. The three transitions could occur at any point over the entire follow-up, but once a person transitioned into an adverse health state, they were considered to stay there until the transition to death or the end of follow-up on Aug 31, 2017. The semi-Markov model took into consideration time since the previous state in the analyses. Analyses were adjusted for age, sex, marital status, race, and birth cohort and presented as HRs with 95% CIs.

In both the survival analyses and multistate models, interval censoring was used because assessment of some adverse health conditions was not continuous; the exact date of onset could have been in the interval between two clinical examinations. We also used inverse probability weighting for missing data,^14^ which involved estimating the probability of being included in the analytic sample (out of the target population) with use of data on sociodemographic factors (age, sex, race, education, height, occupational position, marital status), health behaviours (smoking, alcohol consumption, physical activity, fruit and vegetable consumption), cardiometabolic risk factors (body-mass index, systolic and diastolic blood pressure, cholesterol), mental health from study baseline (1985), and chronic conditions and mortality over the follow-up period (1985 to 2017), as well as interactions with covariates. The inverses of these probabilities were used to weight the analyses. To allow for interval censoring, Weibull distribution was used in both the survival analyses and the multistate models.^15^

Multistate models were performed using the multistate package of R software; all other analyses were done using Stata (version 15). Two-sided p<0·05 was considered to be statistically significant.

### Role of the funding source

The funders of the study had no role in study design, data collection, data analysis, data interpretation, or writing of the report. AD and AS-M had full access to all the data and had final responsibility to submit for publication.

## Results

**Figure 1:**
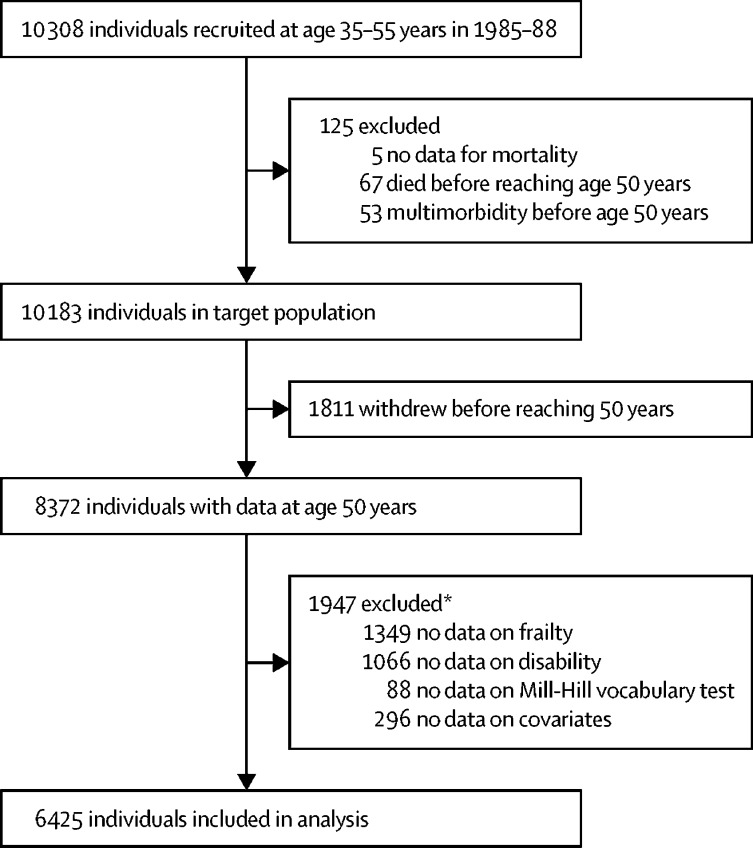
Study profile *Some patients were missing more than one type of data.

Of 10 308 individuals in the Whitehall II cohort study, 8372 had data available at 50 years, of whom 1947 were excluded from this analysis because of missing data on frailty, disability, Mill-Hill vocabulary test, or covariates (figure 1). 6425 individuals were included in our analyses, and our analyses were weighted to reflect the target population of 10 183 participants due to differences in characteristics of these two groups (appendix p 4). Participants were followed up for a median of 23·6 years (IQR 19·6–28·9) and underwent clinical examinations in 1985–88, 1991–93, 1997–99, 2002–04, 2007–09, 2012–13, and 2015–16. The characteristics of participants at age 50 years, as a function of adverse health conditions and mortality at the end of the follow-up, are provided in table 1. Further details of the adverse health conditions of participants are provided in the appendix (p 5); the most frequent component in multimorbidity was coronary heart disease; in frailty was physical inactivity; and in disability was difficulty with dressing.

**Table 1:** Characteristics at age 50 years as a function of multimorbidity, frailty, disability, and mortality status at the end of follow-up (n=6425)

		**Multimorbidity**		**Frailty**		**Disability**		**Mortality**	
		No	Yes	No	Yes	No	Yes	No	Yes
n		4731	1694	4692	1733	5733	692	5814	611
Sex									
	Women	1296 (27·4%)	552 (32·6%)	1203 (25·6%)	645 (37·2%)	1601 (27·9%)	247 (35·7%)	1665 (28·6%)	183 (30·0)
	Men	3435 (72·6%)	1142 (67·4%)	3489 (74·4%)	1088 (62·8%)	4132 (72·1%)	445 (64·3%)	4149 (71·4%)	428 (70·0%)
Ethnicity									
	White	4418 (93·4%)	1494 (88·2%)	4413 (94·1%)	1499 (86·5%)	5294 (92·3%)	618 (89·3%)	5350 (92·0%)	562 (92·0%)
	Other	313 (6·6%)	200 (11·8%)	279 (5·9%)	234 (13·5%)	439 (7·7%)	74 (10·7%)	464 (8·0%)	49 (8·0%)
Marital status									
	Single	1065 (22·5%)	397 (23·4%)	911 (19·4%)	551 (31·8%)	1274 (22·2%)	188 (27·2%)	1300 (22·4%)	162 (26·5%)
	Married or cohabiting	3666 (77·5%)	1297 (76·6%)	3781 (80·6%)	1182 (68·2%)	4459 (77·8%)	504 (72·8%)	4514 (77·6%)	449 (73·5%)
Education level									
	Low	1878 (39·7%)	842 (49·7%)	1884 (40·2%)	836 (48·2%)	2357 (41·1%)	363 (52·5%)	2442 (42·0%)	278 (45·5%)
	Intermediate	1293 (27·3%)	460 (27·2%)	1339 (28·5%)	414 (23·9%)	1585 (27·6%)	168 (24·3%)	1580 (27·2%)	173 (28·3%)
	High	1560 (33·0%)	392 (23·1%)	1469 (31·3%)	483 (27·9%)	1791 (31·2%)	161 (23·3%)	1792 (30·8%)	160 (26·2%)
Occupation									
	Low	526 (11·1%)	309 (18·2%)	465 (9·9%)	370 (21·4%)	694 (12·1%)	141 (20·4%)	739 (12·7%)	96 (15·7%)
	Intermediate	2047 (43·3%)	799 (47·2%)	2033 (43·3%)	813 (46·9%)	2534 (44·2%)	312 (45·1%)	2575 (44·3%)	271 (44·4%)
	High	2158 (45·6%)	586 (34·6%)	2194 (46·8%)	550 (31·7%)	2505 (43·7%)	239 (34·5%)	2500 (43·0%)	244 (39·9%)
Literacy*, mean (SD)		25·2 (4·3)	24·2 (5·1)	25·1 (4·1)	24·4 (5·4)	25·0 (4·4)	23·9 (5·3)	24·9 (4·5)	25·0 (4·4)

Data are n (%), unless otherwise specified.

* Assessed using the Mill Hill vocabulary test.

The median age at occurrence of multimorbidity was 69·1 years (IQR 63·6–74·1), at occurrence of frailty was 69·1 years (IQR 63·1–74·7), at occurrence of disability was 70·5 years (IQR 64·9–75·5), and at occurrence of mortality was 75·4 years (IQR 69·1–79·0). 2877 (44·8%) of 6425 participants had at least one adverse health condition, of whom 1886 (65·6%) had a single adverse health condition and 991 (34·4%) had two or three (appendix p 6). 611 (9·5%) of 6425 participants died, of whom 207 (33·9%) had none of the three adverse health conditions, 236 (38·6%) had one, and 168 (27·5%) had two or three adverse health conditions. Primary causes of death were cancer (295 [48·3%] of 611) and cardiovascular disease (145 [23·7%]; appendix p 7). 1694 (26·4%) of 6425 participants had multimorbidity, of whom 326 (19·2%) died, with a median follow-up of 3·7 years (IQR 1·0–7·3). 1733 (27·0%) of 6425 participants were frail, of whom 195 (11·3%) died, with a median follow-up of 4·4 years (2·3–7·5). 692 (10·8%) of 6425 participants had a disability, of whom 89 (12·9%) died, with a median follow-up of 3·4 years (1·7–6·5). Cancer rather than cardiovascular disease was the primary cause of death in individuals with multimorbidity (50·9% *vs* 19·6%), frailty (36·4% *vs* 25·1%), and disability (36·0% *vs* 19·1%; appendix p 7).

The κ coefficients (education and occupation or literacy κ=0·24, occupation and literacy κ=0·25) did not suggest strong overlap in the socioeconomic status measures. Table 2 shows the associations of socioeconomic indicators at age 50 years with mortality, with the indicators modelled both as categorical and continuous variables. There was no evidence of deviations from linearity, which allowed us to model the socioeconomic status indicators as continuous variables to reflect higher risk in those in the lowest socioeconomic group compared with the highest socioeconomic group. Lower occupational position was associated with increased mortality (HR 1·57 [95% CI 1·19–2·07]). No associations were observed for education and literacy. Occupational position also had the strongest association with multimorbidity, frailty, and disability (appendix p 8).

**Table 2:** Association of socioeconomic indicators at age 50 years with subsequent mortality

		**HR (95% CI)**	**p_non-linearity_**
Education		..	0·13
	High	1 (ref)	..
	Medium	1·22 (0·98–1·52)	..
	Low	1·13 (0·92–1·38)	..
	Education scale*	1·09 (0·89–1·32)	..
Occupation		..	0·93
	High	1 (ref)	..
	Medium	1·24 (1·04–1·49)	..
	Low	1·57 (1·18–2·09)	..
	Occupation scale*	1·57 (1·19–2·07)	..
Literacy†		..	0·37
	High	1 (ref)	..
	Medium	1·00 (0·82–1·22)	..
	Low	1·19 (0·96–1·48)	..
	Literacy scale*	1·02 (0·93–1·12)	..

611 deaths occurred in 6425 participants. Analyses were done using proportional hazards regression with Weibull distribution and inverse probability weighting. Models were adjusted for age, sex, race, marital status, and birth cohort. HR=hazard ratio.

* Categories of socioeconomic status indicators were recoded (0, 0·5, and 1·0) so that when entered as a continuous variable the reported HR corresponded to the increase in risk in the lowest socioeconomic group compared with the highest socioeconomic group.

† Assessed using the Mill Hill vocabulary test.

Sensitivity analyses with varying thresholds to define frailty (two or three of the five components of Fried's frailty score) and disability (one, two, or three Activities of Daily Living) did not affect associations with mortality (appendix p 9), allowing us to retain the threshold of two or more of the condition-specific criteria for all adverse health outcomes. Multimorbidity had the strongest association with mortality (HR 4·12 [95% CI 3·41–4·98]) and disability had the weakest (1·73 [1·34–2·22]; table 3). There was no evidence of stronger associations between adverse health conditions and mortality in the lower socioeconomic status groups. With accumulation of adverse health conditions (none, one, two, or three) as the exposure, there was also no evidence of stronger associations in the lower socioeconomic status groups (appendix p 10).

**Table 3:** Association of multimorbidity, frailty, and disability with subsequent mortality in the total study population and by socioeconomic status

		**Multimorbidity**		**Frailty**		**Disability**	
		HR (95% CI)	p_interaction_*	HR (95% CI)	p_interaction_*	HR (95% CI)	p_interaction_*
Total study population		4·12 (3·41–4·98)	..	2·38 (1·93–2·93)	..	1·73 (1·34–2·22)	..
Education		..	0·74	..	0·29	..	0·16
	High	4·58 (3·27–6·42)	..	3·04 (2·11–4·40)	..	2·58 (1·67–3·99)	..
	Medium	4·22 (2·98–5·96)	..	2·02 (1·37–2·99)	..	1·49 (0·87–2·55)	..
	Low	3·90 (3·00–5·06)	..	2·35 (1·77–3·12)	..	1·62 (1·16–2·25)	..
Occupation		..	0·66	..	0·04	..	0·01
	High	4·52 (3·47–5·89)	..	3·00 (2·21–4·07)	..	2·78 (1·97–3·92)	..
	Medium	4·04 (3·12–5·24)	..	1·82 (1·36–2·43)	..	1·48 (1·02–2·15)	..
	Low	3·63 (2·35–5·61)	..	2·67 (1·65–4·33)	..	1·31 (0·77–2·22)	..
Literacy†		..	0·63	..	0·96	..	0·31
	High	4·15 (3·18–5·40)	..	2·45 (1·83–3·29)	..	1·51 (1·04–2·19)	..
	Medium	3·61 (2·59–5·03)	..	2·38 (1·64–3·45)	..	2·28 (1·47–3·52)	..
	Low	4·49 (3·21–6·28)	..	2·31 (1·61–3·31)	..	1·56 (1·02–2·38)	..

611 deaths occurred in 6425 participants. HRs are compared against having no adverse health condition. Analyses were done using proportional hazards regression with Weibull distribution and inverse probability weighting. Models were adjusted for age, sex, race, marital status, and birth cohort. Participants free of adverse health conditions who withdrew from the study were censored at the data wave that followed their last assessment. HR=hazard ratio.

* The interaction terms tests whether the association between adverse health conditions and mortality differs as a function of socioeconomic status.

† Assessed using the Mill Hill vocabulary test.

**Figure 2:**
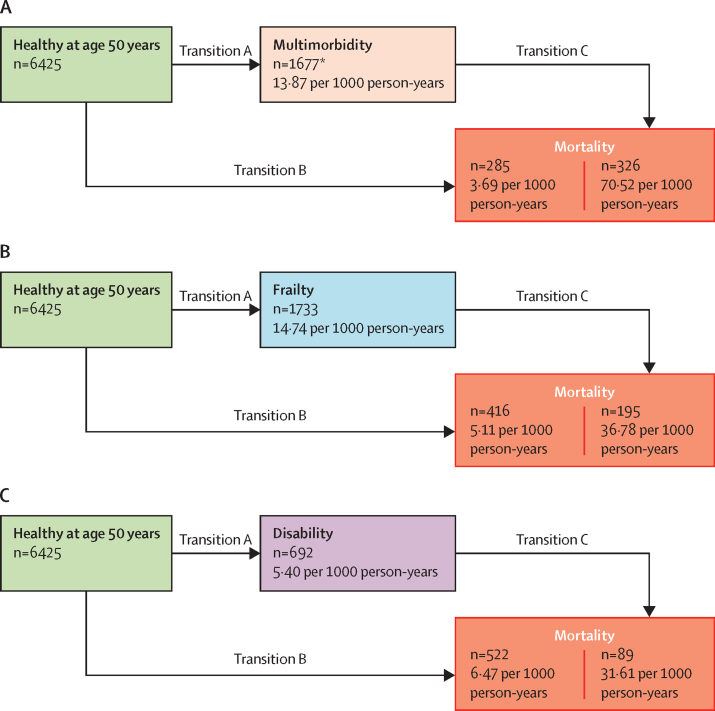
Incidence per 1000 person-years of the transitions from a healthy state at age 50 years to adverse health conditions (multimorbidity, frailty, or disability) and mortality *17 of 1694 participants with multimorbidity died at onset of the second chronic disease. In the analysis of transitions, these participants contribute to transition B rather than transition A.

Figure 2 shows the natural history of disease progression without accounting for differences in socioeconomic status. Of the three adverse health outcomes, the incidence of disability was lowest; mortality was highest in those with multimorbidity. Accounting for socioeconomic status in transitions of health states, there were social inequalities in transition A (from a healthy state to multimorbidity, frailty, or disability) for all socioeconomic status indicators except education in the transition to frailty (table 4). Occupation had the strongest association with transition from a healthy state to multimorbidity (HR 1·54 [95% CI 1·37–1·73]), frailty (2·08 [1·85–2·33]), and disability (1·44 [1·18–1·74]).

**Table 4:** Multistate models for the transitions from a healthy state to adverse health condition (multimorbidity, frailty and disability) and mortality

	**n/N**	**Education, HR (95% CI)**	**Occupation, HR (95% CI)**	Literacy*, HR (95% CI)
**Transition to mortality via multimorbidity**				
A (healthy to multimorbidity)	1677†/6425	1·24 (1·13–1·35)	1·54 (1·37–1·73)	1·11 (1·07–1·14)
B (healthy to mortality)	285/6425	0·94 (0·67–1·34)	2·02 (1·18–3·44)	0·89 (0·74–1·07)
C (multimorbidity to mortality)	326/1677†	1·03 (0·89–1·20)	1·14 (0·94–1·39)	0·99 (0·94–1·05)
**Transition to mortality via frailty**				
A (healthy to frailty)	1733/6425	1·08 (0·99–1·18)	2·08 (1·85–2·33)	1·05 (1·01–1·09)
B (healthy to mortality)	416/6425	1·26 (1·05–1·50)	1·82 (1·45–2·30)	1·10 (1·03–1·19)
C (frailty to mortality)	195/1733	0·92 (0·76–1·11)	0·96 (0·76–1·22)	0·96 (0·90–1·02)
**Transition to mortality via disability**				
A (healthy to disability)	692/6425	1·29 (1·11–1·50)	1·44 (1·18–1·74)	1·21 (1·14–1·27)
B (healthy to mortality)	522/6425	1·15 (1·01–1·31)	1·39 (1·17–1·65)	1·02 (0·97–1·08)
C (disability to mortality)	89/692	0·69 (0·50–0·95)	0·90 (0·59–1·36)	1·00 (0·90–1·12)

Analyses were done using multistate models (three states) with interval censored data, and with Weibull distribution and inverse probability weighting. Models were adjusted for sex, race, marital status, and birth cohort, and in transition C, age at the adverse health condition. Categories of socioeconomic status indicators were recoded (0, 0·5, and 1·0) so that when entered as a continuous variable the reported HR corresponded to the increase in risk in the lowest socioeconomic group compared with the highest socioeconomic group. HR=hazard ratio.

* Assessed using the Mill Hill vocabulary test.

† 17 of 1694 participants with multimorbidity died at onset of the second chronic disease. In the analysis of transitions, these participants contribute to transition B rather than transition A.

Social inequalities were also observed in transition B (from a healthy state to mortality in those without an adverse health condition), which might be because cancer was the primary cause of death in individuals who died without multimorbidity (45·3%), frailty (53·8%), and disability (50·4%; appendix p 7). We found no evidence of excess mortality in the lower socioeconomic groups in those with multimorbidity, frailty, or disability (transition C). Age at onset of adverse health conditions did not modify associations (range of p values for interactions between socioeconomic status indicators and age at onset: 0·13 to 0·96). Sensitivity analyses on transition C, using any of the three adverse health conditions as the transition state with 404 deaths in 2877 participants, also showed education (HR 0·89 [95% CI 0·78–1·02), occupation (1·13 [0·95–1·34]), and literacy (0·99 [0·94–1·04) not to be associated with mortality in this transition (appendix p 11).

## Discussion

In this study, we analysed temporal progression from a healthy state at age 50 years to adverse health conditions (multimorbidity, frailty, disability) and subsequent mortality. We found that social inequalities are generated before, rather than after, the onset of these conditions. Therefore, socioeconomic status affects the risk of multimorbidity, frailty, and disability but does not affect the risk of mortality in individuals with these conditions. Our approach differed from mediation models, in which the goal is to estimate the extent to which adverse health conditions explain social inequalities; instead, we investigated how socioeconomic status affects the onset and progression of adverse health conditions. Results from our study show social inequalities in the cause rather than the prognosis of the studied conditions.

Of the three adverse health conditions in our study, multimorbidity had the strongest association with mortality; the strength of this association was similar across socioeconomic status groups. 175 (28·6%) of 611 deaths in our study were in individuals with only multimorbidity and a further 151 deaths (24·7%) in those with multimorbidity and frailty, disability, or both. The importance of multimorbidity is increasingly being recognised,^16^, ^17^ particularly at older ages,^17^ although no clear consensus has been reached on the precise number or nature of diseases that should be included in the definition of multimorbidity. Similarly, the concept of frailty is increasingly promoted as a simple measure of health status of older adults,^18^ but definitions vary across studies, as reflected in alternative conceptualisations, such as the Frailty Index.^19^ In general terms, frailty is the result of cumulative decline in multiple physiological systems, reflecting a state of heightened susceptibility to environmental stressors.^11^, ^18^ Disability is widely used in research on ageing, is often measured using difficulties in daily living,^1^ and it predicts future health outcomes.^20^ Although we found no consistent evidence of heterogeneity across socioeconomic groups in the association between adverse health conditions and mortality, the associations were generally slightly stronger in the high occupation and high education groups. This counterintuitive finding could be explained by functional limitations being more normative in groups of low socioeconomic status or by better reporting of these conditions in groups of high socioeconomic status.

Strong evidence shows that social inequalities exist in multimorbidity,^17^ frailty,^21^ and disability;^22^ all three conditions have been associated with greater risk of mortality.^2^, ^3^, ^4^ Analysis of data from the World Health Surveys^1^ suggests that trends in loss of functioning and disability over the life course might be improving, but chronic disease patterns appear to be worsening. Our findings suggest that multimorbidity is a particularly important public health concern because of its high prevalence, supported by findings in previous studies,^17^ and its strong association with mortality. Although the prevalence of multimorbidity increases with age, it is not uncommon for an individual to experience multimorbidity before old age.^17^ Thus, better monitoring of multimorbidity and timely interventions might help to improve population health; such monitoring is feasible because individuals will have contact with the health-care provider for management of the first non-fatal chronic disease (ie, before a first chronic disease progresses to multimorbidity). Our selection of nine chronic diseases is based on their importance for mortality; among individuals without multimorbidity in this study, the majority of deaths (73·7%) were due to cardiovascular disease or cancer, which are both included on the list of nine diseases.

The traditional view of disease progression is that it follows a series of stages: risk factors, disease or condition, loss of function, disability, and death.^23^ This temporal sequence was not seen in our study, because the median age at onset of multimorbidity, frailty, and disability was similar. Furthermore, nearly two-thirds of the individuals with adverse health conditions experienced only one of the three conditions. Using a follow-up starting at age 50 years, we considered multimorbidity, frailty, and disability with subsequent mortality in the same study; most previous research has not examined all three adverse health conditions, therefore their relative importance for mortality and social inequalities in mortality could not be assessed. Our results identify multimorbidity as the most pertinent prevention target to improve population health and prolong life expectancy. Even when all adverse health conditions were considered together in sensitivity analyses, we found no evidence of social inequalities in transitions to mortality in individuals with one or more of these conditions.

In this study, occupation reflected education, salary, and social status, and it was strongly associated with all three adverse health conditions. The use of three socioeconomic status indicators allows comparisons to be made between them and suggests that comprehensive measures of socioeconomic status are more strongly associated with health conditions than measures of single aspects of socioeconomic status. Long life expectancies will require individuals to self-manage health for long periods, which has led to an interest in health literacy:^24^ an individual's capacity to obtain, process, and understand health information (in contrast to indicators of socioeconomic circumstances that reflect access to resources). In this study, literacy was not associated with mortality and weakly associated with adverse health conditions. Of note, none of the socioeconomic status indicators affected the risk of mortality after the onset of multimorbidity, frailty, or disability. Two previous studies reported similar findings in relation to comprehensive measures of socioeconomic circumstances and frailty: one study based in China,^25^ and the other based on the Honolulu-Asia Aging Study.^26^ In our previous study, we used a narrow definition of multimorbidity, cardiometabolic multimorbidity, and found that health behaviours, rather than socioeconomic status, play a major role in the transition from multimorbidity to mortality.^27^

The strengths and limitations of this study must be considered. The study had a large sample size, multiple measures of socioeconomic status, long follow-up to allow analyses of the natural history of health conditions using suitable methods, and availability of complete data on health conditions. Although multiple sources were used to ascertain health conditions, data on some conditions could have been missing because we did not have access to emergency care records. However, chronic conditions are unlikely to have been treated only in emergency care, and the resulting imprecision in date of onset is accounted for in the interval censoring used in our analyses. A further strength is the consideration of missing data using inverse probability weighting; the availability of data on occupational position and mortality for everyone in the target population (1830 deaths in 10 183 individuals) allowed us ensure that the association in this population was similar to that in our sample after inverse probability weighting.

The primary limitation of this study is that our findings are likely to apply only to high-income countries with universal health care, where the onset of disease or poor health triggers the involvement of health and social care systems. A further limitation is the use of an occupational cohort study, in which participants tend to be healthier than those in the general population, and which does not allow for comparison of prevalence rates with studies based on the general population. However, we have previously shown estimates from the Whitehall II cohort study to be similar to those reported in general population-based studies; therefore the study population is unlikely to be a source of bias in associations between risk factors and disease.^28^ Although occupation in our study reflects income, it is not a measure of family wealth, living conditions, or other financial difficulties. Similarly, the measure of literacy used in the study is only a proxy of health literacy and some of its associations with health could be poorly estimated. Finally, because no gold-standard definitions exist for the adverse health conditions, we set the threshold for all three conditions as meeting two out of a list of criteria, which is the method primarily used in studies on multimorbidity, and these thresholds could have affected the results. The complex relationships between multimorbidity, frailty, and disability over time, including reversal from these conditions, was not considered in our analyses because of computational complexity and limited statistical power.

Changes in behaviours and improvements in the treatment of major diseases have led to increases in life expectancy in recent decades. Our analysis of the transitions from adverse health conditions to mortality show that multimorbidity is an important target to improve population health and reduce social inequalities in mortality. Health care systems that are organised around single-system illness will need to be restructured to reflect the multiorgan dysfunction experienced by older adults. Our results highlight the importance of prevention, either via management of risk factors or screening, and effective treatment of early stages of disease, to avoid social inequalities in mortality and improve population health.

## Data sharing

De-identified individual participant data from the Whitehall II study are available on request.
