# Supplementary material for: Social inequalities in multimorbidity, frailty, disability, and transitions to mortality: a 24-year follow-up of the Whitehall II cohort study
Source: Lancet Public Health. 2019 Dec 16;5(1):e42–50. doi: 10.1016/S2468-2667(19)30226-9 (PMC7098476; doi:10.1016/S2468-2667(19)30226-9)
Supplement: Supplementary appendix [file mmc1.pdf]

THE LANCET
Public Health

Supplementary appendix

This appendix formed part of the original submission and has been peer reviewed. We post it as supplied by the authors.

Supplement to: Dugravot A, Fayosse A, Dumurgier J, et al. Social inequalities in multimorbidity, frailty, disability, and transitions to mortality: a 24-year follow-up of the Whitehall II cohort study. *Lancet Public Health* 2019; published online Dec 11. https://doi.org/10.1016/S2468-2667(19)30226-9.

Supplementary Appendix

Social inequalities in multimorbidity, frailty, disability, and transitions to mortality: a 24-year follow-up of the Whitehall II cohort study

Appendix Text

Components of Fried's Frailty Scale

1. Low level of physical activity was denoted by an energy expenditure of <383 kcal/week (men) and 270 (women), estimated from a 20-item questionnaire on frequency and duration of participation in activities (e.g., running, cycling, other sports, housework, and gardening activities). A metabolic equivalent (MET) value was assigned to each activity to calculate energy expenditure for each participant.
2. Slow walking speed was assessed using time taken to walk 8 feet (2·4 meters) at usual pace, the cut-offs were based on results for a 15-ft (4·6 m) walking test. Slow walking speed was defined as time to walk 8-ft ≥3·73 s for men with height ≤173 cm or women with height ≤159 cm, or ≥3·20 s for men with height >173 cm or women with height >159 cm.
3. Grip strength was measured using a Smedley hand grip dynamometer. Sex and body mass index (BMI) specific thresholds were used. In men grip strength was poor when ≤29 kg for BMI≤24 kg/m^2, ≤30 kg for BMI 24·1–28 kg/m^2, and ≤32 kg for BMI >28 kg/m^2. In women grip strength was poor when ≤17 kg for BMI≤23 kg/m^2, ≤17·3 kg for BMI 23·1-26 kg/m^2, ≤18 kg for BMI 26·1-29 kg/m^2, and ≤21 kg for BMI>29 kg/m^2.
4. Weight loss is defined as being either unintentional or as a proportion of body weight lost over the previous year; as weight was measured at clinical examination every five years, we utilized a cut-off of 10% of loss in body weight as used in the Women's Health Aging Study-I.[1]
5. Exhaustion was defined using two items of the Center for Epidemiology Studies-Depression (CES-D) scale: "I felt that everything I did was an effort in the last week" and "I could not get going in the last week".[2] Participants were deemed to fulfil this criteria if their response was "occasionally or moderate amount of the time (3-4 days)" or "most or all of the time (5-7 days)".

References

1. Boyd CM, Xue QL, Simpson CF, et al. Frailty, hospitalization, and progression of disability in a cohort of disabled older women. *Am J Med* 2005;118(11):1225-31. doi: 10.1016/j.amjmed.2005.01.062 [published Online First: 2005/11/08]

2. Bouillon K, Sabia S, Jokela M, et al. Validating a widely used measure of frailty: are all sub-components necessary? Evidence from the Whitehall II cohort study. *Age (Dordr)* 2013;35(4):1457-65. doi: 10.1007/s11357-012-9446-2 [doi]

eTable 1. Population characteristics in 1985-1988 (Whitehall II cohort baseline) and mortality status at the end of follow-up in analytic sample and target population.[a]

	Analytic sample	Target population [a]
N	6,425	10,183
Men, N (%)	4,577 (71·2)	6,816 (66·9)
Non-white, N (%)	513 (8·0)	1,019 (10·1)
Single, N (%)	1,462 (22·8)	2,609 (25·7)
Low education, N (%)	2,720 (42·3)	4,841 (47·5)
Low occupation, N (%)	835 (13·0)	2,299 (22·6)
Mortality, N (%)	611 (9·5)	1,860 (18·3)

[a]A total of 10,308 participants were recruited to the study in 1985-1988. The target population was used in inverse probability weighting to reflect the baseline population of the study, excluding participants who could not be traced for mortality (N=5), died (N=67) or had multimorbidity (N=53) before reaching age 50.

eTable 2. Components of multimorbidity, frailty, and disability.[a]

Multimorbidity		Frailty		Disability	
**Component**	**N (%)**	**Component**	**N (%)**	**Component**	**N (%)**
Diabetes	617 (9·6)	Physically inactive	1,423 (22·1)	Bathing	471 (7·3)
Coronary Heart Disease	764 (11·9)	Slow walking speed	453 (7·1)	Dressing	593 (9·2)
Stroke	131 (2·0)	Poor grip strength	819 (12·7)	Toileting	274 (4·3)
Chronic Obstructive Pulmonary Disease	122 (1·9)	Weight loss	285 (4·4)	Transferring	325 (5·1)
Depression	484 (7·5)	Exhaustion	824 (12·8)	Feeding	143 (2·2)
Arthritis	612 (9·5)			Walking	245 (3·8)
Cancer	530 (8·2)				
Dementia	96 (1·5)				
Parkinson's disease	53 (0·8)				
**Total**	**1,694 (26·4%)**		**1,733 (27·0%)**		**692 (10·8%)**

[a]Percentage calculated using total study sample, N=6,425.

eTable 3. Overlap in adverse health conditions in the study sample, N=6,425.

	**Total population N=6,425**	**Mortality N=611**
**Adverse Health Conditions**	**N**	**N**
No adverse health conditions	3,548	207
One or more adverse health condition	2,877	404
**Only one adverse health condition**		
Multimorbidity	845	175
Disability	166	52
Frailty	875	9
**Two adverse health conditions**		
Multimorbidity and Disability	133	25
Frailty and Disability	159	17
Multimorbidity and Frailty	465	88
**All three adverse health conditions**	**234**	**38**

eTable 4. Causes of death, with and without multimorbidity, frailty, and disability over the follow-up.

Cause of death		Multimorbidity		Frailty		Disability	
	**All**	**Yes**	**No**	**Yes**	**No**	**Yes**	**No**
Cardiovascular disease	145 (23·7%)	64 (19·6%)	81 (28·4%)	49 (25·1%)	96 (23·1%)	17 (19·1%)	128 (24·5%)
Cancer	295 (48·3%)	166 (50·9%)	129 (45·3%)	71 (36·4%)	224 (53·8%)	32 (36·0%)	263 (50·4%)
Respiratory disease	42 (6·9%)	21 (6·4%)	21 (7·4%)	24 (12·3%)	18 (4·3%)	11 (12·4%)	31 (5·9%)
Other	129 (21·1%)	75 (23·0%)	54 (18·9%)	51 (26·2%)	78 (18·8%)	29 (32·6%)	100 (19·2%)
**Total**	**611**	**326**	**285**	**195**	**416**	**89**	**522**

eTable 5. Association of socioeconomic indicators at age 50 with adverse health conditions (multimorbidity, frailty, and disability).[a]

	Multimorbidity[b] HR (95% CI)	Frailty HR (95% CI)	Disability HR (95% CI)
**Education**			
High	1 (ref)	1 (ref)	1 (ref)
Medium	1·21 (1·05, 1·40)	0·92 (0·80, 1·06)	1·08 (0·86, 1·36)
Low	1·26 (1·10, 1·44)	1·07 (0·95, 1·21)	1·30 (1·06, 1·59)
P non-linearity	*0·19*	*0·07*	*0·60*
Education scale[c]	1·23 (1·08, 1·40)	1·10 (0·97, 1·25)	1·32 (1·07, 1·62)
**Occupation**			
High	1 (ref)	1 (ref)	1 (ref)
Medium	1·36 (1·21, 1·52)	1·36 (1·21, 1·53)	1·21 (1·00, 1·45)
Low	1·66 (1·40, 1·97)	2·20 (1·85, 2·62)	1·66 (1·26, 2·18)
P non-linearity	*0·33*	*0·11*	*0·44*
Occupation scale[c]	1·67 (1·42, 1·97)	2·19 (1·84, 2·62)	1·65 (1·25, 2·16)
**Literacy[d]**			
High	1 (ref)	1 (ref)	1 (ref)
Medium	1·15 (1·01, 1·30)	0·78 (0·69, 0·89)	1·15 (0·94, 1·40)
Low	1·34 (1·17, 1·54)	1·00 (0·87, 1·15)	1·38 (1·11, 1·71)
P non-linearity	*0·89*	*<0·001*	*0·84*
Literacy scale[c]	1·14 (1·07, 1·20)	1·06 (0·99, 1·14)	1·21 (1·11, 1·33)

Numbers multimorbidity: 1694/6425; frailty: 1733/6425; disability: 692/6425.
[a]Analyses (adjusted for age, sex, race, marital status, and birth cohort) using proportional hazards regression with Weibull distribution with inverse probability weighting and interval censoring.
[b]Multimorbidity defined as 2 or more of the following chronic conditions: diabetes, coronary heart disease, stroke, chronic obstructive pulmonary disease, depression, arthritis, cancer, dementia, and Parkinson's disease.
[c]Categories of socioeconomic indicators were recoded (0, 0·5, and 1) so that when entered as a continuous variable the reported hazard ratio corresponded to increased risk in the lowest compared to the highest socioeconomic group.
[d]Assessed using the Mill Hill vocabulary test.

eTable 6. Association of frailty and disability, with varying thresholds, with mortality.[a]

	N	HR (95% CI)
**Frailty criteria**		
2 or more out of 5	1733/6425	2·38 (1·93, 2·93)
3 or more out of 5	453/6425	2·84 (2·19, 3·68)
**Disability criteria**		
1 or more ADL	1401/6425	1·93 (1·58, 2·36)
2 or more ADL	692/6425	1·73 (1·34, 2·22)
3 or more ADL	390/6425	2·11 (1·59, 2·80)

[a]Analyses (adjusted for age, sex, race, marital status, and birth cohort) using proportional hazards regression with Weibull distribution with inverse probability weighting.

eTable 7. Association of the number of adverse health condition (time varying) with subsequent mortality in the total study population and analysis stratified by SES.[a]

	0 adverse health condition<br>HR (95% CI)	1 adverse health condition<br>HR (95% CI)	2 adverse health conditions<br>HR (95% CI)	3 adverse health conditions<br>HR (95% CI)
**Total sample**	**1 (ref)**	**2·38 (1·93, 2·93)**	**1·73 (1·34, 2·22)**	**4·55 (3·67, 5·65)**
**Education**				
High	1 (ref)	3·89 (2·50, 6·04)	7·48 (4·62, 12·12)	15·93 (6·37, 39·83)
Medium	1 (ref)	2·98 (1·98, 4·50)	5·33 (3·13, 9·09)	4·11 (1·25, 13·52)
Low	1 (ref)	2·96 (2·07, 4·24)	4·44 (2·96, 6·67)	6·36 (3·42, 11·82)
p for interaction[b]		***0·58***	***0·23***	***0·14***
**Occupation**				
High	1 (ref)	3·05 (2·14, 4·36)	7·29 (4·97, 10·69)	9·84 (4·63, 20·91)
Medium	1 (ref)	3·50 (2·52, 4·86)	3·99 (2·67, 5·97)	5·87 (3·08, 11·21)
Low	1 (ref)	2·25 (1·11, 4·57)	4·67 (2·37, 9·18)	5·40 (1·49, 19·56)
p for interaction[b]		***0·51***	***0·07***	***0·53***
**Literacy[c]**				
High	1 (ref)	2·83 (1·99, 4·02)	5·98 (4·09, 8·76)	5·50 (2·50, 12·14)
Medium	1 (ref)	3·17 (2·11, 4·78)	4·36 (2·63, 7·22)	8·97 (4·41, 18·24)
Low	1 (ref)	3·60 (2·25, 5·75)	5·16 (3·05, 8·73)	4·72 (1·38, 16·07)
p for interaction[b]		***0·71***	***0·58***	***0·54***

[a]Analyses (adjusted for age, sex, race, marital status, and birth cohort) using proportional hazards regression with Weibull distribution and with inverse probability weighting. Participants free of adverse health conditions who dropped out of the study were censored at the data wave that followed their last assessment.

[b] The interaction terms tests whether the association between the number of adverse health conditions and mortality differs as a function of socioeconomic indicators.

[c]Assessed using the Mill Hill vocabulary test.

eTable 8. Multi-state models for the transitions from a healthy state to any adverse health condition (multimorbidity, frailty, or disability) and mortality.[a]

TRANSITIONS	N event/ N total	Education[b] HR (95% CI)	Occupation[b] HR (95% CI)	Literacy[b,c] HR (95% CI)
**Transition to mortality via any of the 3 adverse health conditions**				
A (Healthy to Any adverse health condition)	2877/6425	1.17 (1.09, 1.26)	1.91 (1.74, 2.09)	1.09 (1.06, 1.13)
B (Healthy to Mortality)	207/6425	2.06 (1.27, 3.36)	1.81 (0.78, 4.20)	1.05 (0.84, 1.31)
C (Any adverse health condition to Mortality)	404/2877	0.89 (0.78, 1.02)	1.13 (0.95, 1.34)	0.99 (0.94, 1.04)

[a]Analyses using three states multi-state models with interval censored data; analyses undertaken using Weibull distribution and inverse probability weighting.
Analysis adjusted for sex, race, marital status, birth cohort, and age at the adverse health outcome in the transition C.
[b]Categories of socioeconomic indicators were recoded (0, 0.5, and 1) so that when entered as a continuous variable the reported hazard ratio corresponded to increased risk in the lowest compared to the highest socioeconomic group.
[c]Assessed using the Mill Hill vocabulary test.
